# Interactions Between Infectious Foodborne Viruses and Bacterial Biofilms Formed on Different Food Contact Surfaces

**DOI:** 10.1007/s12560-022-09534-z

**Published:** 2022-08-27

**Authors:** Marie-Josée Gagné, Tony Savard, Julie Brassard

**Affiliations:** Agriculture and Agri-Food Canada, Saint-Hyacinthe Research and Development Centre, 3600 Casavant Boulevard West, Saint-Hyacinthe, QC J2S 8E3 Canada

**Keywords:** Foodborne viruses, Biofilms, Interaction, Persistence, Food contact surfaces

## Abstract

Bacterial biofilms contribute to contamination, spoilage, persistence, and hygiene failure in the food industry, but relatively little is known about the behavior of foodborne viruses evolving in the complex communities that make up biofilm. The aim of this study was to evaluate the association between enteric viruses and biofilms on food contact surfaces. Formed biofilms of mono- and multispecies cultures were prepared on glass, stainless steel, and polystyrene coupons and 10^5^ pfu/ml of murine norovirus, rotavirus, and hepatitis A virus were added and incubated for 15 min, 90 min, and 24 h. The data obtained clearly demonstrate that the presence of biofilms generally influences the adhesion of enteric viruses to different surfaces. Many significant increases in attachment rates were observed, particularly with rotavirus whose rate of viral infectious particles increased 7000 times in the presence of *Pseudomonas fluorescens* on polystyrene after 24 h of incubation and with hepatitis A virus, which seems to have an affinity for the biofilms formed by lactic acid bacteria. Murine norovirus seems to be the least influenced by the presence of biofilms with few significant increases. However, the different factors surrounding this association are unknown and seem to vary according to the viruses, the environmental conditions, and the composition of the biofilm.

## Introduction

Enteric viruses, such as HuNoV, hepatitis A and E viruses, and rotavirus, represent a public health real burden as they are the leading cause of foodborne illness outbreaks. HuNoV alone leads to 699 million cases of acute gastroenteritis, 219,000 deaths, $4.2 billion in direct health care costs, and $56.2 billion in lost productivity globally each year (Bartsch et al., 2016). Fresh produce, meats and processed and ready-to-eat foods, provide an ideal transmission route for these viruses. Unlike most microbiological agents, viruses cannot replicate on food and therefore contamination levels do not increase during processing or storage. However, very few infectious viral particles are required to induce disease, and enteric viruses are known to be resistant to commonly used disinfectants, as well as being stable in a variety of harsh environments including on surfaces, thus enabling their transmission through the food chain (Koopmans & Duizer, 2004).

Food processing environments are prime locations for the development of surface-associated microbial communities known as biofilms (Brooks & Flint, 2008). These complex structures protect the microorganisms that compose them. This molecular arrangement forms a 3D network composed of extracellular polymeric substances (EPS), such as polysaccharides, biopolymers, proteins, nucleic acids, and lipids (Garrett et al., 2008). Food industry biofilms represent complex polymicrobial communities where a wide variety of bacterial species can persist on various types of equipment and non-porous surfaces such as stainless steel, polypropylene, rubber, wood, and glass present and used in the food processing industries (Bridier et al., 2015). It is well known that biofilms contribute to contamination, persistence, and hygiene failure in the food industry since they can harbor pathogenic bacteria such as *Listeria monocytogenes*, *Campylobacter jejuni*, *Bacillus* spp. or *Escherichia coli* but also bacteria responsible for food spoilage (Brooks & Flint, 2008). The decontamination of food contact surfaces is an important issue for the industry, and several studies have been published in the last decade suggesting strategies such as chemical treatments, food surface modification with nanoparticles, enzymatic disruption, and plant essential oil to control or prevent biofilm formation on food surfaces (Galie et al., 2018) and reduce the risk to consumers. Other approaches have been suggested such as the development of a protective and competitive biofilm on food and contact surfaces composed by lactic acid bacteria (LAB) to avoid pathogen growth by secreting antimicrobial molecules (bacteriocins, organic acids, biosurfactants) and control the formation of the biofilm from *Listeria monocytogenes*, *Escherichia coli* O157:H7, and *Salmonella Typhimurium* (Alvarez-Ordonez &Briandet, 2017; Gomez et al., 2016).

In vivo, enteric viruses and bacteria have always coexisted and evolved in the gastrointestinal microbiota and may target the same ecological niches, but it is only recently that we have become interested in the possible relationships between these two types of microorganisms. An in-depth review on the observed interactions between enteric bacteria and eukaryotic viruses was recently published by Berger and Mainou (2018). The authors reported studies demonstrating, for instance, that the presence of certain bacterial species in the intestinal tract of the hosts promoted poliovirus, norovirus, rotavirus, and murine norovirus infections (Jones et al., 2014; Moore & Jaykus, 2018; Sullender & Baldridge, 2018); that poliovirus and norovirus have the ability to adhere to bacterial cell wall components including lipopolysaccharide (LPS), peptidoglycan, extracellular polymeric substances, and surface polysaccharides (Amarasiri & Sano, 2019; Robinson et al., 2014); and that, in the presence of certain bacterial components, the thermostability of Reovirus is enhanced (Berger et al., 2017). Also, recent studies have reported that norovirus and rotavirus have the ability to bind certain bacterial strains such as *E. cloacae, E. coli* Nissle, and some lactic acid bacteria (LAB) (*L. rhamnosus, B. bifidum*, etc.) which, in addition to being present in the intestinal tract, can also be found in biofilms in food processing industries and in competitive biofilms (Cai et al., 2018; Chen et al., 2017).

There is limited information surrounding the occurrence and survival of foodborne viruses in bacterial biofilms in the food sector and their potential role in the viral dissemination in this environment (Dawley & Gibson, 2019). Nevertheless, a first study demonstrating the incorporation and interactions between poliovirus and biofilms present in a wastewater treatment plant was published in 1997 (Quignon et al., 1997). Other studies, also conducted in water treatment plants, have reported the presence of poliovirus, Cryptosporidium parvum, Giardia lamblia, and a multitude of phages associated with biofilms colonizing surfaces within a drinking water distribution system, as well as enteroviruses and noroviruses associated with biofilms from wastewater tanks (Helmi et al., 2008; Skraber et al., 2009). Very recently, the viral composition of biofilms from wastewater treatment plants was identified by metagenomic analyses, demonstrating the abundance and diversity of viral families found within these biofilms (Petrovich et al., 2019). It can be speculated that interactions between enteric viruses and bacteria may promote easier entry of viral particles into the biofilm, creating a potential reservoir of pathogenic viruses. However, there are virtually no data on the presence of infectious particles associated with biofilms formed on surfaces since the data reported were obtained by detection of viral genomic material. Therefore, the aim of the present study was to examine the interactions between infectious foodborne viruses and biofilms formed on surfaces used in the agri-food sector. Murine norovirus (surrogate for human norovirus), hepatitis A virus, and rotavirus were used to determine viral adhesion to spoilage and LAB biofilms in static conditions on three different food contact surfaces (glass, stainless steel, polystyrene) over time.

## Materials and Methods

### Bacterial Strains, Growth Media, and Conditions

*Lactobacillus plantarum* ATCC 10241, *Leuconostoc pseudomesenteroides* ATCC 12291, and *Lactobacillus rhamnosus* RW9595M strains were propagated in lactobacilli MRS broth (Difco, MD, USA), while *Pseudomonas fluorescens* ATCC 13525 was propagated in Tryptic Soy broth (Difco) and all bacteria were incubated for 20 h at 30 °C. They were subcultured twice at 1:10 in 10-ml broth before use from BHI-glycerol tubes (stored at − 80 °C) for all experiments. Bacterial viability was determined by plating on MRS agar and TSA agar, respectively, and bacterial counts were expressed as colony-forming units (CFU) per ml. After the last 20 h of incubation, the bacterial counts for *P. fluorescens, L. plantarum, L. pseudomesenteroides, and L. rhamnosus* obtained were 2 × 10^9^ CFU/ml, 9.5 × 10^8^ CFU/ml, 2.5 × 10^9^ CFU/ml, and 3.0 × 10^8^ CFU/ml, respectively.

### Viruses and Cell Lines

Hepatitis A virus (HAV) cytopathic strain HM-175 was propagated on FRhK-4 cell line ATCC CRL-1688 obtained from the American Type Culture Collections (ATCC, Massanas, VA, USA) according to Bozkurt et al. (Bozkurt et al., 2015) on confluent cell monolayers. Murine norovirus strain 1 (MNV-1) and rotavirus WA were propagated on RAW 264.7 cell line ATCC TIB-71 as described by Gonzalez-Hernandez et al. (Gonzalez-Hernandez et al., 2012) and MA-104 cell line ATCC CRL-2378.1 as described by Arnold et al. (Arnold et al., 2009), respectively. Virus titers were determined using cell culture plaque assay and were expressed as plaque-forming units (PFU) per ml. Virus stocks were stored at − 80 °C until use.

### Biofilm Formation and Virus Inoculation

**Fig. 1:**
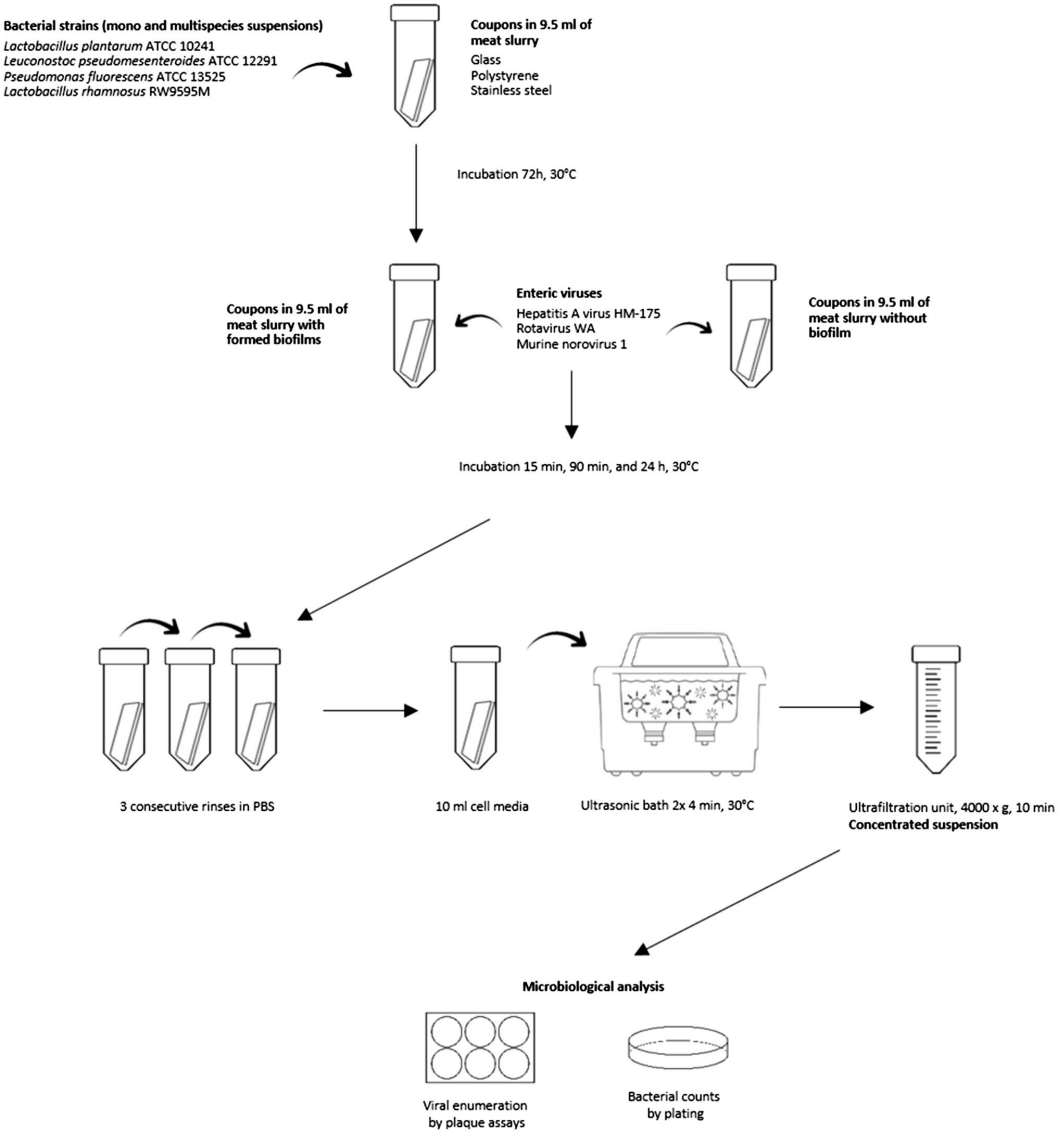
Schematic representation of experimental design

Figure 1 illustrates the experimental design that was developed to evaluate the interactions between biofilms formed on surfaces and enteric viruses. Biofilm formation was conducted in 50-ml Falcon tubes with sterile coupons of glass (18 mm × 18 mm, Fisher scientific, Waltham, MA), stainless steel 316 (15 mm × 15 mm) and polystyrene (18 mm × 18 mm; Fisher scientific), and 9.5 ml in meat slurry (10% beef juice + 3% enzymatic meat digest + 1.5% dextrose + 2.206% sodium citrate dibasic) according to Lapointe et al. (2019) with an addition of 500 µl of each bacterial strain monoculture and multispecies (prepared with a 1:4 ratio of each monoculture suspension) suspensions. Coupons were incubated for 72 h at 30 °C with bacterial suspensions to create a biofilm on those surfaces in static condition. Subsequently, viruses were added into the tube at final concentration of 10^5^ pfu/ml to the formed biofilm, and tubes were agitated and re-incubated at 30 °C for 15 min, 90 min, and 24 h in order to allow virus–bacteria interactions and viral particle adhesion. After the incubations, coupons were washed extensively three times in 5 ml PBS (Wisent) by dipping using sterile forceps to remove non-adherent cells and then transfer them into 10 ml DMEM-5 (MNV-1), M199 serum-free (RoV), or DMEM/F12 (HAV) (Fig. 1). Tubes were vortexed at maximum speed for 30 s. Then, biofilms and viruses were removed from surfaces by sonication in an ultrasonic bath (VWR B2500A-DTH, 40 Hz) with 2 × 4 min at 30 °C and an agitation in between the two treatments. Bacteria–viral suspensions were transferred into Amicon Ultra-15 centrifugal filter units (EMD Millipore 100 kDa) and were concentrated by centrifugation for 10 min at 4000×*g*.

To assess biofilm formation on the three selected food contact surfaces, the bacterial cell population density, expressed in log_10_CFU/cm^2^, was determined for each bacterial strain on each type of coupons after the incubations. Samples from concentrated suspensions were serially diluted in peptone water and spread onto TSA agar for enumeration of *P. fluorescens* and MRS agar for enumeration of *Lactobacillus* and *Leuconostoc* strains. Colony counting was performed after 20 h of incubation of the plates at 30 °C.

In order to evaluate the impact of biofilms on viral adhesion to surfaces, viral counts obtained from the three food contact surfaces covered with biofilms were compared to viral counts obtained from the biofilm-free food contact surfaces. Clean coupons of the three types of material were placed separately in 9.5 ml of meat slurry and the three different viruses were added to a final concentration of 10^5^ pfu/ml followed by incubations of 15 min, 90 min, and 24 h at 30 °C. Recovery of the viral particles was performed as shown in Fig. 1 and the assays were all performed in triplicate. Viral enumerations of infectious RoV, HAV, and MNV were performed as described in the following sections from the concentrated suspensions obtained from both the biofilm-covered and biofilm-free coupons.

### Viral Enumeration of Rotavirus

Rotavirus enumeration was conducted according to the Arnold et al. protocol (Arnold et al., 2009) with some modifications. Briefly, MA-104 cells were grown in six-well plates with M199 media (supplemented with 5% FBS, 100 U/ml penicillin, 100 µg/ml streptomycin) and incubated at 37 °C with 5% CO_2_ for 3 days to obtain a confluence. In order to activate the viruses, samples were incubated with 10 µg/ml of trypsin (Sigma, Oakville, ON, Canada) at 37 °C for 1 h. Then, samples were diluted in tenfold dilutions in serum-free M199 media and 500 µl was plated per well in duplicate and incubated for 1 h at 37 °C with 5% CO_2_ on a rocking platform. The inoculums were removed from wells and 3 ml of warmed EMEM 2X overlay medium (containing EMEM 2X without phenol red with 5% FBS, 5 mM HEPES, 50 U/ml penicillin, 50 µg/ml streptomycin, 2 mM l-Glutamine, 2% SeaPlaque agarose, and 0.5 µg/ml of trypsin) were added to each well and plates were incubated at 37 °C with 5% CO_2_ for 4 days. Plaque visualization was possible with the addition of a second overlay containing EMEM supplemented with 2% SeaPlaque agarose and 25 µg/ml of neutral red after a 24-h incubation at 37 °C with 5% CO_2_.

### Viral Enumeration of Hepatitis A virus

HAV quantification was done as described by Bozkurt et al. (2015) with slight modifications. Enumerations of viable particles were conducted using confluent FRhK-4 cells in 6-well plates with DMEM/F12 media supplemented with 10% FBS, 100 U/ml penicillin, and 100 µg/ml streptomycin (Wisent Bioproducts, Boucherville, QC, Canada) and incubated at 37 °C with 5% CO_2_ for 3 days. Samples were tenfold diluted in DMEM/F12 (supplemented with 2% FBS, 100 U/ml penicillin, and 100 µg/ml streptomycin) before being inoculated at 500 µl per well in duplicate. Plates were incubated for 90 min at 37 °C with 5% CO_2_ on a rocking platform. Then, inoculums were removed from wells, and cells were overlaid with 2 ml warmed EMEM overlay medium [4 mM l-glutamine, 0.22% sodium bicarbonate, 0.20% non-essential amino acids, 100 U/ml penicillin, and 100 µg/ml streptomycin and 1% SeaPlaque agarose (Lonza, Mississauga, ON, Canada)]. Plates were incubated at 37 °C with 5% CO_2_ for 7 days. After incubation, cells were stained with 2 ml/well of 0.33% neutral red solution in PBS at 37 °C with 5% CO_2_ for 1 h. Neutral red solution was removed and plaques were counted in plaque-forming units/ml (PFU/ml).

### Viral Enumeration of Murine Norovirus

MNV-1 viability was determined according to Gonzalez-Hernandez et al. (Gonzalez-Hernandez et al., 2012) with confluent RAW 264.7 cells with DMEM-10 (DMEM supplemented with 10% fetal bovine serum (FBS), 10 mM HEPES, 100 U/ml penicillin 100 µg/ml streptomycin, 1 mM non-essential amino acids, and 2 mM l-Glutamine) in six-well plates (24 h at 37 °C with 5% CO_2_). Samples were diluted in tenfold dilutions with DMEM-5 (DMEM with 5% FBS) and 500 µl was added per well in duplicate. Viral attachment was done with gentle agitation at 21 °C for 1 h on a rocking platform. Afterward, samples were removed and 2 ml of warmed MEM 2X overlay medium (EMEM 2X without phenol red with 5% FBS, 5 mM HEPES, 50 U/ml penicillin 50 µg/ml streptomycin, 2 mM l-Glutamine, and 2% SeaPlaque agarose) was added on cells. Plates were incubated at 37 °C with 5% CO_2_ for 48 h. Plaque counts in PFU/ml were done after staining with 2 ml/well of 0.33% neutral red solution in PBS and an incubation at 37 °C with 5% CO_2_ of 2 h.

### Statistical Analysis

Statistical analyses were performed using GraphPad Prism v6 (GraphPad Software Inc, San Diego, CA, USA). Means and standard deviations from three independent experiments were determined. Error bars indicate standard deviation, and Student’s *t* test was used to compare recovered viral counts originating from surfaces without biofilm with recovered viral counts from surfaces with biofilms. Values of *P* < 0.05 were considered statistically significant.

## Results and Discussion

### Biofilm Formation on Food Contact Surfaces

**Fig. 2:**
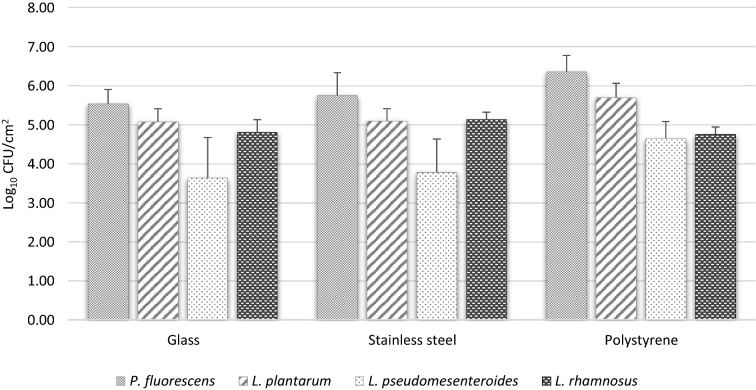
Density of viable biofilm cell populations of *P. fluorescens, L. plantarum, L. pseudomesenteroides, and L. rhamnosus* in each biofilm formed on 3 different food contact surface coupons after an incubation of 72 h at 30 °C in meat slurry media. Values are expressed as means

In this study, four bacterial strains (*L. plantarum*, *L. pseudomesenteroides*, *P. fluorescens*, and *L. rhamnosus*) able to form biofilms and found in the agri-food sector (Lapointe et al., 2019) were inoculated in monoculture and multispecies combination in the presence of glass, stainless steel, and polystyrene coupons. Biofilm formation was conducted in a meat slurry media to mimic the composition of organic material found in the meat industry for 3 days at 30 °C in static condition and at the same concentration for each bacterial strain. The averages of the density of viable biofilm cell populations on the coupons collected after 3 days were 4.76 log_10_ CFU/cm^2^, 4.94 log_10_ CFU/cm^2^, and 5.43 log_10_ CFU/cm^2^ for glass, stainless steel, and polystyrene, respectively (Fig. 2). Those results showed that coupons made of polystyrene seem to promote better coverage of the surface by bacterial biofilms compared to glass and stainless steel. Polystyrene is known to be a hydrophobic material, and it has been shown that this characteristic can influence the attachment of bacteria to surfaces and promote biofilm formation (Di Ciccio et al., 2015). Of the 4 tested bacteria, *P. fluorescens* strain showed the higher density of viable cell populations for the 3-food contact selected surfaces with a cell density of 5.88 log_10_ CFU/cm^2^ which is significantly different from the lowest density of 4.02 log_10_ CFU/cm^2^ observed for *L. pseudomesenteroides* (Fig. 2)*.* The observed population densities are slightly lower than those observed by Lapointe et al. with the same bacterial strains (Lapointe et al., 2019). However, the biofilm formation conditions were different, and it is recognized that factors such as temperature, incubation time, agitation, and type and roughness of surfaces can influence the ability of bacterial strains to form biofilms which could explain the results obtained in this study (Rodriguez-Melcon et al., 2018). Furthermore, the optimized conditions used in this study have allowed the establishment of a reasonable cell population density and coverage for each surface and with each bacterial strain after 72 h in order to evaluate the ability of the biofilms formed to capture foodborne enteric viruses.

### Viral Attachment to Food Contact Surfaces

It is clearly recognized that fomites and surfaces play a prominent role in the transmission of enteric viruses and contribute to their dissemination (Barker et al., 2001; Vasickova et al., 2010). It is also recognized that viruses have the ability to persist and adhere to different types of environmental surfaces through nonspecific interactions and the forces implicated in the attachment of viral particles have been described as a multiplex interaction between electrostatic, hydrophobic, and Van der Waals (Armanious et al., 2016; Gerba, 1984). The pH, isoelectric point, surface roughness, liquid, structure of the virus itself, and presence of organic matter are also factors that can influence the adhesion/attachment of viral particles. However, the mechanisms underlying these interactions are still not clearly defined, and it was therefore important in this study to evaluate the attachment of viruses alone to the different selected food contact surfaces in order to characterize the impact of the presence of biofilm on the viral adhesion. Viral attachment of RoV, MNV, and HAV on biofilm-free coupons included in Table 1 shows, as expected, that these viruses can adhere to the three clean food contact surfaces selected. Up to 2.60 log_10_ PFU/ml of infectious virus particles of MNV and HAV were recovered from the stainless steel coupons after 24 h of incubation. The virus counts in this study were determined by plaque assay on tissue cultures and expressed in PFU/ml, as it is the titer of infectious viral particles recovered from the surfaces with or without biofilm, not a population density.

**Table 1:** Infectious viral particles recovered from biofilm-free surface coupons after different incubations at 30 °C in meat slurry media

Virus	Viral titer, Log_10_ PFU/ml^a^								
	Glass			Stainless steel			Polystyrene		
	15 min	90 min	24 h	15 min	90 min	24 h	15 min	90 min	24 h
Rotavirus	2.96 ± 0.38	2.54 ± 0.09	0.24 ± 0.33	2.32 ± 0.40	2.07 ± 0.33	0.0 ± 0.0	2.67 ± 0.52	2.07 ± 0.05	0.0 ± 0.0
Hepatitis A virus	2.96 ± 0.10	2.56 ± 0.24	2.63 ± 0.23	2.60 ± 1.30	2.24 ± 0.55	2.96 ± 0.17	2.44 ± 0.28	2.44 ± 0.12	2.47 ± 0.23
Murine norovirus	2.28 ± 0.70	2.42 ± 0.30	2.62 ± 0.30	2.25 ± 0.07	2.35 ± 0.49	2.95 ± 0.21	2.63 ± 0.52	2.63 ± 0.33	2.22 ± 0.31

^a^Values are expressed as means ± standard deviations

Also, the data demonstrated that only 15 min of contact time can be sufficient to allow viruses to interact with and adhere to the surfaces, with an average of 1.76 log_10_ PFU of infectious viral particles recovered per coupon. The amount of attached viral particles generally seemed to be stable over time, except for RoV, in which a decrease in adhesion was observed after 24 h (Table 1). Infectious RoV particles tend to detach from the surface and be found free in the meat slurry (data not shown). The detachment of rotavirus particles from surfaces compared to the stable of adhesion of HAV and MNV could be explained by the dissimilarity of their respective isoelectric point values (IPV). The IPV can influence the adsorption of viral particles to solid surfaces as well as their release into the medium (Dowd et al., 1998). In the presence of a low viral isoelectric point (net negative charge), an increase in adsorption to solid surfaces has been observed; whereas, when the value is close to neutrality or greater, adsorption levels are lower and viruses tend to be released. The viral isoelectric point seems to have an influence by increasing the strength of the charges. The IPV determined for RoV is 8.0, while the IPVs for MVN and HAV are approximately 4.7 and 2.8, respectively (Bolton et al., 2013; Michen & Graule, 2010). At a naturally neutral or slightly acidic pH value as in the case of the meat slurry (pH of 6.60) used in this study, MNV and HAV will retain a negative charge, while rotavirus will have a positive charge, which could impact the adsorption of the virus to different solid surfaces. It has also been reported that the presence of organic matter in water and soil can block the attachment of rotavirus compared to HuNoV (Gamazo et al., 2020).

### Interactions Between Formed Biofilms on Food Contact Surfaces and Enteric Viruses

The presence of interactions between viruses and bacteria that promote infection is now undeniable, and the interest in studying the mechanisms underlying them is growing (Stern et al., 2019). These studies are mainly focused on basic microbiology, i.e., on pathogens, their replication mechanisms, and their interactions with the host. However, as very limited published studies examine the interactions of microorganisms relevant to applied agricultural and agri-food sciences (Moore & Jaykus, 2018), it seemed important to evaluate the interaction between certain enteric viruses and bacterial biofilms formed on contact surfaces or different surfaces considering that both entities can be found in the same food sector environments and that they can compromise product safety. Moreover, the use of foodborne enteric viruses and cultivable surrogates in this study provides crucial data on their persistence and infectivity when associated with biofilms, and information that is essential to better understand the risk they pose if associated with environmental biofilms (Von Borowski & Trentin, 2021).

### Interactions Between Biofilms and Rotavirus

**Fig. 3:**
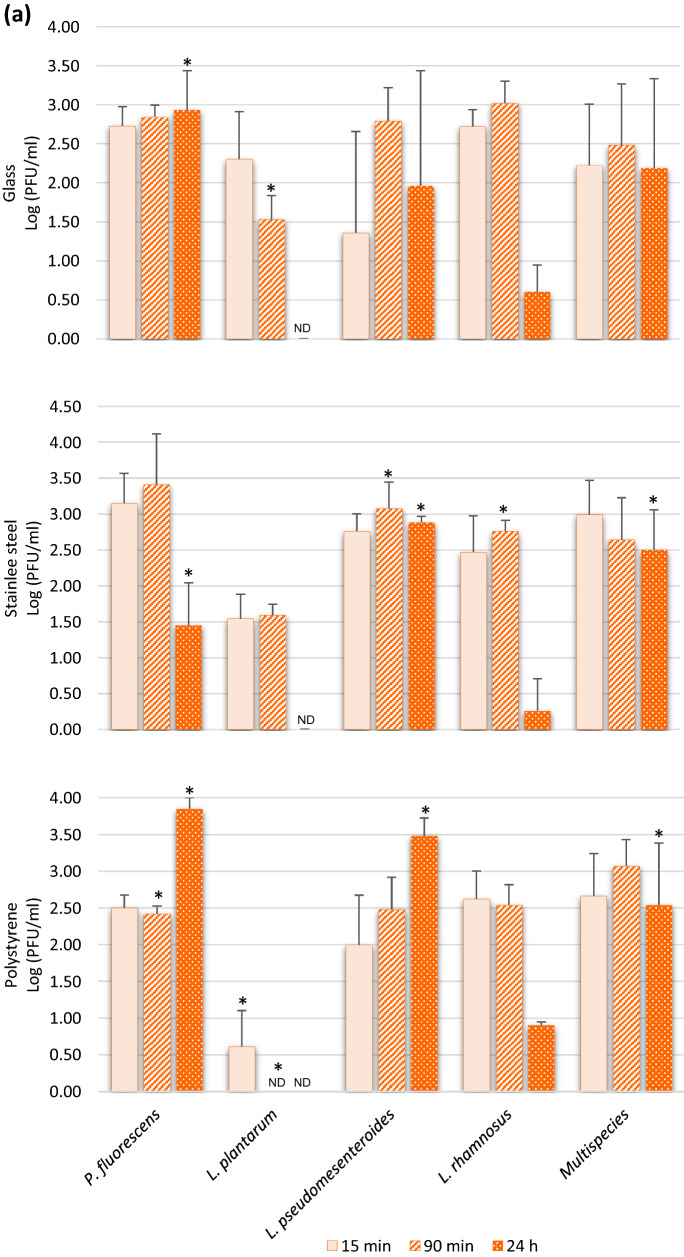

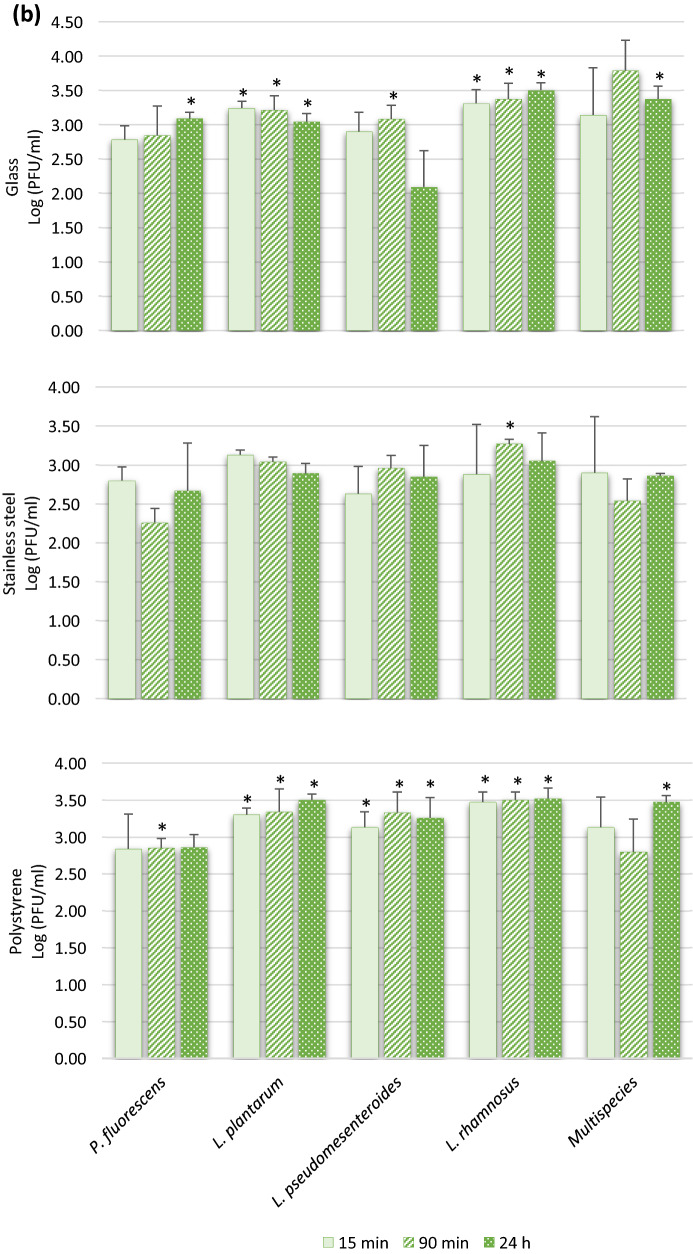

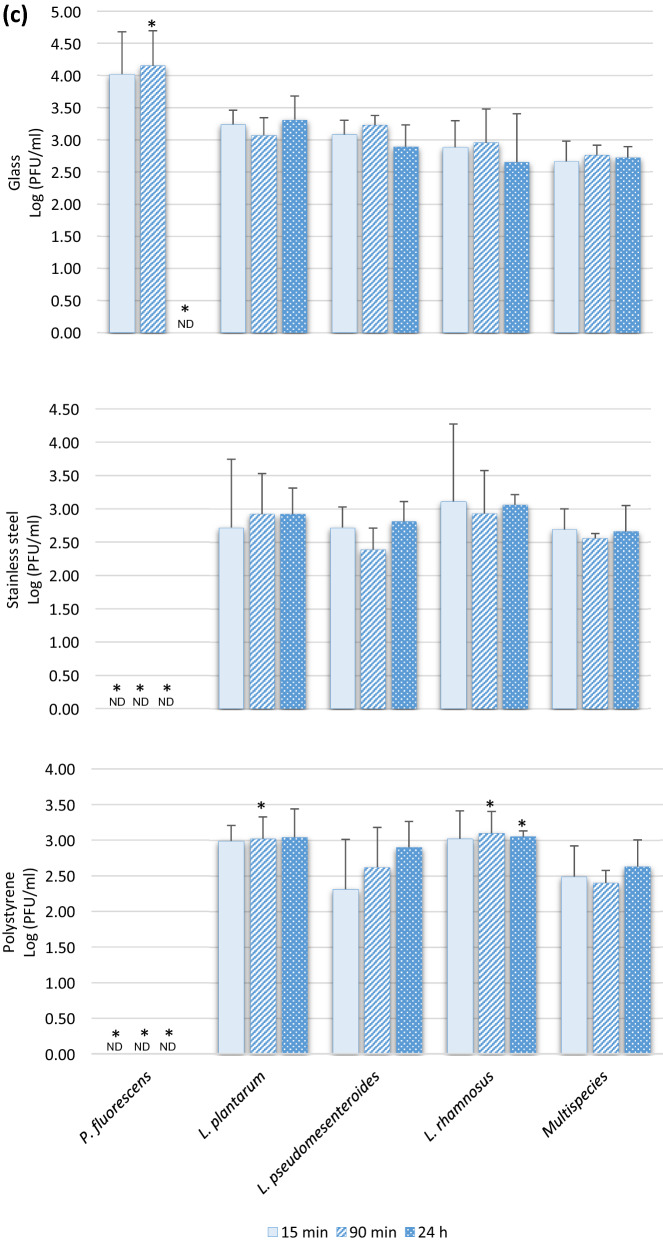
Recovered infectious viral particles of rotavirus (RoV) (panel **a**), Hepatitis A virus (HAV) (panel **b**), and murine norovirus (MNV) (panel **c**) from biofilms formed on 3 different food contact surfaces in monoculture and multispecies culture after incubation periods of 15, 90 min, and 24 h. The viral counts were determined by plaque assays on tissue cultures. Statistical analyses were performed using GraphPad Prism v6 (GraphPad Software Inc, San Diego, CA, USA). Means and standard deviations from three independent experiments were determined. Error bars indicate standard deviation and Student’s t test was used to compare recovered viral counts originating from surfaces without biofilm with recovered viral counts from surfaces with biofilms. *P* values < 0.05 were considered statistically significant and indicated with a (*)

The infectious viral particles recovered from the different food contact surfaces with formed biofilms are shown in Fig. 3. The presence of biofilms on these surfaces seems to promote the adhesion of enteric viruses since several viral concentrations are significantly higher than in the absence of biofilm (Table 1). RoV is the virus in which a significant increase in adhesion of infectious particles (*P* < 0.05) was observed in the presence of formed biofilms, especially after 24 h of incubation, on the stainless steel and the polystyrene surfaces (Fig. 3a). After 24 h of incubation in the absence of biofilm, a very small amount of rotavirus viral particles was recovered from the surfaces (Table 1). However, attachment rates of up to 7000 and 3000 times more viral particles were found attached to the biofilm formed on the polystyrene by *P. fluorescens and L. pseudomesenteroides*, respectively (Fig. 3a). In addition, results showed that the multispecies biofilm significantly promotes the adhesion (*P* < 0.05) of RoV infectious viral particles on the two of the three different food contact surfaces tested in this study. In contrast, the colonization of a surface by *L. rhamnosus* does not seem to favor the adhesion of the RoV, since it is in the presence of this biofilm that the virus showed the least increase of adhesion on the three selected surfaces. Interestingly, results from randomized controlled trials in patients have confirmed that administration of *L. rhamnosus* GG (LGG) as a probiotic has beneficial effects on rotavirus-induced diarrhea, such as reducing symptoms and duration of illness (Ahmadi et al., 2015). In addition to interference with viral replication, more focused studies have shown that LGG prevents epithelial damage and ameliorates rotavirus-induced diarrhea by modulating immune cells, such as dendritic cells and inflammatory cytokines (Jiang et al., 2017; Liu et al., 2013). Thus, the colonization of certain biotic and abiotic surfaces by *L. rhamnosus* may interfere and reduce the association/attachment with the RoV viral particle.

### Interactions Between Biofilms and Hepatitis A Virus

To the author’s knowledge, there are no data in the literature on the possible association between HAV and bacterial components, bacteria of the microbiota, or biofilms. The data collected in this study show that when surfaces were coated with formed biofilms, HAV adhesion increased significantly (*P* < 0.05), particularly on glass and polystyrene coated with LAB, especially with *L. plantarum* and *L. rhamnosus* and to a lesser extent with *L. pseudomesenteroides* (Fig. 3b). In addition, the increase in adhesion was observed from 15 min of incubation and was maintained over time in comparison with rotavirus, where the significant increase was observed mainly after 24 h. According to the World Health Organization (WHO), 1.4 million new cases of hepatitis A are reported each year worldwide, resulting in nearly 7000 deaths (WHO). Although there is no legislation on the presence of HAV in environmental matrices and food at the global level despite the variation in hygiene standards and disease prevalence in different regions of the world, some countries, including Canada, have adopted measures for the surveillance of HAV in some food products. In developed countries, fresh and processed (frozen) fruits and vegetables are generally involved in HAV foodborne outbreaks, and the sources of viral contamination of those products are primarily infected food handlers, contaminated water, and surfaces (Bozkurt et al., 2015). The virus also has the ability to persist in hostile environments on surfaces, water, and food (Leblanc et al., 2019), and the levels of viral contamination in real life conditions may be low and focal and, therefore, difficult to detect on food products. The results also show that the biofilm formed by *L. rhamnosus* on a 6.48 cm^2^ surface allows the adhesion of 347 infectious HAV particles, which is 8 times more than on the surface alone. As the infectious dose of HAV is very low (10–100 viral particles), the potential transfer of a very small amount of infectious viral particles associated with bacterial-formed biofilm during processing, either by contact or by biofilm dispersion on a food item, could be sufficient to trigger a foodborne infection. It has also been suggested that colonization of food surfaces by some type of LAB could be exploited to mitigate pathogens and spoilage microorganisms, reducing food poisoning, product spoilage and the use of chemical agents (Arena et al., 2017). However, the results set out in this study may suggest that a purposeful immobilization of LAB as a pathogen biocontrol on targeted surfaces, such as food contact surfaces, could favor the capture of infectious viral particles under certain conditions and potentially become a reservoir of foodborne viruses, especially HAV. Moreover, the results emphasize the importance of further studies both in lab scale and under real conditions to understand the mechanisms underlying these interactions and the potential risk they pose to food safety.

### Interactions Between Biofilms and Murine Norovirus

The presence of biofilms formed by LAB and multispecies culture on the different food contact surfaces did not generally promote or reduce (*P* ˃ 0.05) the presence of more MNV particles, used as surrogate for HuNoV, since the numbers of infectious particles recovered are similar to those found on the surfaces without biofilm (Table 1), i.e., on average 2.80 vs 2.38 PFU/ml, 2.88 vs 2.30 PFU/ml, and 2.88 vs 2.59 PFU/ml for 15 min, 90 min, and 24 h, respectively, for all surfaces combined (Fig. 3c). Only a few significant increases (*P* ˂ 0.05) in adhesion were found, and only on the polystyrene colonized by the two Lactobacillus biofilms after 90 min and 24 h (Fig. 3c). It is also possible that the bacterial strains and certain experimental conditions, such as static vs bioreactors; incubation time, medium, and pH used in this study did not favor the attachment of MNV to the formed biofilms. It has been shown that the HuNoV-bacteria binding efficiency can be impacted by the richness of the culture media (Almand et al., 2017). Furthermore, the biofilm-producing bacteria used in this study are not recognized to express HBGA-like substances and NoVs, including MNV, are known for their ability to bind HBGA-like substances present in tissues and environmental material such as leafy green and oyster digestive tissue and expressed in some intestinal bacteria (Amarasiri & Sano, 2019). However, norovirus strains have different HBGA recognition profiles and bacterial strains expressing these substances are infrequently identified and wide variations in their expression of HBGA-like substances have been observed (Li et al., 2015). Other structures, compounds or mechanisms that still unknown, are probably involved in the bacteria–noroviruses binding since this takes place with bacteria that do not express HBGA-like substances (Almand et al., 2017). However, it would be interesting for future studies to include *E. cloacae* since it is present in processing plants, has the ability to form biofilms, and expresses HBGA-like substances (Cai et al., 2018). These data could allow us to evaluate the impact on the level of adhesion of MNV to biofilms formed on surfaces in absence and presence of this putative receptor. Furthermore, it has been shown that HBGA–NoV interaction with bacterial components, or with oyster digestive tissues, allows the viruses to escape abiotic stresses, be more stable, and survive (Amarasiri & Sano, 2019). It can be hypothesized that a NoV viral particle associated with an *E. cloacae* biofilm formed on a food contact surface could possibly be “protected” and less susceptible to the actions of washing and disinfection in a food processing plant. However, future studies are needed to confirm this hypothesis.

Interestingly, after 24 h in the presence of a biofilm formed by *P. fluorescens* on the three tested surfaces, no infectious viral particles of MNV were detected by plaque assay (Fig. 3c). Nevertheless, the detection of MNV RNA by RT-qPCR was performed on biofilms recovered from *P. fluorescens*, and the virus RNA is indeed present at relatively high concentrations, i.e., 2.4 × 10^4^ of genomic copies/coupon (data not shown) and would theoretically have allowed titration on the cell line. It would appear that the virus has been inactivated or that essential receptors for infection of MNV have been blocked by components present in the *P. fluorescens* biofilm, but the mechanism behind this observation is not known. Also, this phenomenon was not observed with HAV and RoV in the presence of *P. fluorescens* and with MNV in contact with the other bacteria, which excludes an issue with the recovery method. It would be interesting to confirm if this phenomenon is reproduced with other enteric viruses, particularly with HuNoV, different strains, and genotypes and to identify the substance produced by *P. fluorescens* responsible for the drastic decrease in infectivity.

## Conclusion

The present study, to the best of author’s knowledge, is one of the first to demonstrate that the presence of biofilms formed on food contact surfaces with spoilage bacteria and LAB could promote the attachment of infectious foodborne viruses under certain conditions. However, the use of three viruses from different species—hepatitis A virus, rotavirus, and murine norovirus—clearly demonstrates that they do not have the same behavior nor the same binding abilities under the same experimental conditions. Furthermore, it is important to be able to evaluate the infectious potential of viral particles associated with biofilms, and for the moment, molecular detection does not allow this. It is therefore essential to further explore the mechanisms involved in these interactions in order to better understand the role that biofilms may play as a reservoir of foodborne viruses, in the dispersion and possible contamination of food products and in the persistence of viruses in the food sector environment, since interactions with biofilms could protect viruses from desiccation, make them less sensitive to washing and disinfection, and prolong their survival in the environment.

## Data Availability

The datasets generated during and/or analyzed during the current study are available from the corresponding author on a reasonable request.
